# Guideline-Concordant Sedative and Analgesia Use in Critically Ill Patients Receiving Sustained Neuromuscular Blockade

**DOI:** 10.1097/CCE.0000000000001370

**Published:** 2026-01-26

**Authors:** Megan E. Feeney, Ava E. Canova, Anica C. Law, Allan J. Walkey, Nicholas A. Bosch

**Affiliations:** 1 Department of Pharmacy, Boston Medical Center, Boston, MA.; 2 Department of Medicine, The Pulmonary Center, Boston University Chobanian & Avedisian School of Medicine, Boston, MA.; 3 Department of Healthcare Delivery and Population Sciences, University of Massachusetts Chan Medical School—Baystate, Springfield, MA.; 4 Division of Health Systems Science, Department of Medicine, University of Massachusetts Medical School, Worcester, MA.; 5 Evans Center for Implementation and Improvement Science, Department of Medicine, Boston University Chobanian & Avedisian School of Medicine, Boston, MA.

**Keywords:** analgesia, critical illness, intensive care units, neuromuscular blocking agent, sedatives

## Abstract

**OBJECTIVES:**

Given significant harms of awake paralysis, guidelines recommend combination sedation and analgesia during neuromuscular blockade. In this study, we sought to describe guideline-concordant use of parenteral sedation and analgesia during sustained neuromuscular blockade among adults with critical illness.

**DESIGN:**

Multicenter, retrospective cohort study.

**SETTING:**

ICUs in 871 hospitals across the United States.

**PATIENTS:**

Adult patients admitted to an ICU between 2016 and 2022 who received invasive mechanical ventilation and at least one neuromuscular blocking agent (NMBA) on the same calendar day.

**INTERVENTIONS:**

None.

**MEASUREMENTS AND MAIN RESULTS:**

We used charge codes to identify use of parenteral sedatives (propofol, fospropofol, lorazepam, midazolam, diazepam, etomidate, phenobarbital, pentobarbital, and ketamine), and analgesics (fentanyl, hydromorphone, morphine, and ketamine) for each patient-day with neuromuscular blockade, and then categorized patient-days as guideline concordant or not. A total of 363,382 patient-days (among 104,984 hospitalizations) were included in the final cohort. Guideline-concordant sedation and analgesia were used concurrently in 345,660 patient-days (95.1%); only sedation was used in 15,618 patient-days (4.3%), only analgesia was used in 1,348 patient-days (0.4%), and neither sedation nor analgesia was used in 756 patient-days (0.2%). Most included hospitals (856 [98.3%]) used both sedation and analgesia on greater than or equal to 50% of patient-days; however, ten hospitals (1.1%) used only sedation on greater than or equal to 50% of patient-days, and 4 (0.6%) had no predominant sedation and analgesia strategy; however, 42.3% of the variation in guideline-concordant practice was attributable to residual unexplained clustering by hospital after accounting for demographics, severity of illness, and hospital characteristics.

**CONCLUSIONS:**

Our findings suggest sedation and analgesia practices with NMBA use in adult ICUs are generally guideline-concordant but require corroboration using more precise, quantitative medication data. Practice variation between hospitals is potentially concerning and warrants further investigation targeting adequacy of sedation and analgesia during NMBA use and assessing the clinical impact of guideline-discordant practices.

**KEY POINTS**

**Question**: Are U.S. ICUs using guideline-concordant sedation and analgesia in adults receiving sustained neuromuscular blockade?

**Findings**: Guideline-concordant sedation and analgesia were used concurrently with continuous neuromuscular blockade in 345,660 patient-days (95.1%); however, guideline-concordant practice was highly variable between hospitals and was associated with several clinical and demographic characteristics.

**Meaning**: Our results suggest that most sedation and analgesia use during continuous neuromuscular blockade is guideline-concordant, but there remains a need to reduce potentially inappropriate variation in sedation and analgesia use at the hospital level.

Neuromuscular blocking agents (NMBAs) are used in ICUs for muscle paralysis by inhibiting the transmission of nerve impulses at the neuromuscular junction. NMBAs lack sedative and analgesic properties; patients not receiving adequate sedation during neuromuscular blockade may experience overwhelming panic, pain, and loss of control (1, 2) and awareness with paralysis has been reported in up to 3.4% of emergency department and ICU patients (3, 4). To avoid recall, the 2016 Society of Critical Care Medicine Guidelines (5) suggest administration of both sedation and analgesia before and during sustained neuromuscular blockade. Single-center studies (6, 7) have identified gaps between NMBA initiation and administration of sedation and analgesia in critically ill patients receiving neuromuscular blockade; building on these studies, we used a large multicenter cohort to quantify guideline-concordant use of parenteral sedation and analgesia during sustained neuromuscular blockade among critically ill patients in the United States.

## METHODS

We used the Premier PINC AI Healthcare Database (Premier, Charlotte, NC; from April 2016 to September 2022), a multicenter, enhanced claims-based database including ~25% of U.S. hospitalizations with characteristics similar to the American Hospital Association Database (8). PINC AI includes *International Classification of Diseases*, 10th Revision codes and itemized hospital charge codes with resolution to the calendar day and minimal missing data (8).

We identified adult patients admitted to ICUs who received invasive mechanical ventilation (IMV) and at least one NMBA (cisatracurium, atracurium, vecuronium, or rocuronium) on the same calendar day. For each patient, we included consecutive days where both IMV and NMBA were used to best approximate sustained neuromuscular blockade (5). We excluded the first day of IMV and NMBA use and days during which the patient underwent surgery or general anesthesia. The unit of analysis was the patient-day.

For each patient-day with neuromuscular blockade, we determined use of parenteral sedatives (propofol, fospropofol, lorazepam, midazolam, diazepam, etomidate, phenobarbital, pentobarbital, and ketamine) and analgesics (fentanyl, hydromorphone, morphine, and ketamine) using medication charge codes. Dexmedetomidine was not included due to inadequacy in achieving deep sedation or sufficient analgesia when used as a single agent. We then categorized each patient-day into the following practices: both sedation and analgesia (i.e., guideline-concordant practice), sedation-only, analgesia-only, and neither sedation nor analgesia. Charge codes included medication formulations that could last for more than 24 hours when administered at typical infusion rates. Thus, we extrapolated the expected duration of each formulation using average rates of sedation and analgesia infusions reported in a clinical trial evaluating neuromuscular blockade in acute respiratory distress syndrome (9) (**eTables 1** and **2**, https://links.lww.com/CCX/B597). This meant that patients without sedative or analgesic charge codes on a day of NMBA might still meet our definition of guideline-concordant practice if the patient had charge codes on prior days for formulations that could potentially still be in use on the day of NMBA.

We calculated the number and percentage of patient-days with each sedation and analgesia practice overall and stratified by hospital. We then fitted a hierarchical logistic regression model (10) for the outcome (dependent variable) of guideline-concordant practice (yes/no) with fixed effects for demographics (age, sex, race, Hispanic ethnicity), discharge year, principal diagnosis (categorized using the top five diagnoses among patients for each practice pattern with all other diagnoses labeled as “other” to improve model convergence), acute organ dysfunction (defined by Angus et al [11]) present at admission (12), and hospital characteristics (Census region, teaching, urban, and safety net status) and a random intercept term for the hospital. We reported the intraclass correlation coefficient (ICC), the percentage of variation in guideline-concordant practice attributable to the hospital of admission after accounting for fixed effects, and adjusted odds ratios (aORs [95% CIs]) for the association between fixed effects and guideline concordance. The modeling cohort was limited to hospitals with at least 25 patient-days meeting inclusion criteria to improve model convergence.

This study was deemed not human subjects research by the Boston University Institutional Review Board (H-41795) and no review was necessary. R (Version 4.0.5; R Foundation for Statistical Computing, Vienna, Austria) was used for analyses.

## RESULTS

Among 11,000,518 ICU patient-days with IMV, NMBAs were administered on 1,958,640 patient-days. A total of 363,382 patient-days (among 104,984 hospitalizations) were included in the final cohort. Cisatracurium was the most frequently used NMBA (*n* = 234,485 [64.5%]). Guideline-concordant sedation and analgesia were used in 345,660 (95.1%) patient-days; nonconcordant sedation-only use occurred in 15,618 patient-days (4.3%), analgesia-only use in 1,348 patient-days (0.4%), and neither sedation nor analgesia in 756 patient-days (0.2%). The mean age was 55 years (sd, 14 yr; **eTable 3**, https://links.lww.com/CCX/B597). The most common principal diagnoses were sepsis (*n* = 141,564 [39.0%]), COVID-19 (*n* = 132,261 [36.4%]), and acute respiratory failure (*n* = 14,714 [4.0%]). Similar rates of guideline-concordant practices were seen for patient-days with a COVID-19 diagnosis (229,303 [95.6%]) and without a COVID-19 diagnosis (116,357 [94.2%]). A total of 233,823 patient-days (64.3%) resulted in hospital death or discharge to hospice.

The median number of sedatives (interquartile range [IQR], 1–2; range, 0–6) and analgesics (IQR, 1–1; range, 0–4) used per patient-day was 1. Midazolam (60.6% of patient-days) was the most frequently used sedative, followed by propofol (59.4% of patient-days). The most frequently used analgesic was fentanyl (76.1% of patient-days). Dexmedetomidine was used in 51,442 patient-days (14.2%); if dexmedetomidine was categorized as a sedative, only 223 (0.06%) additional patient-days would be considered guideline-concordant.

**Figure 1.**
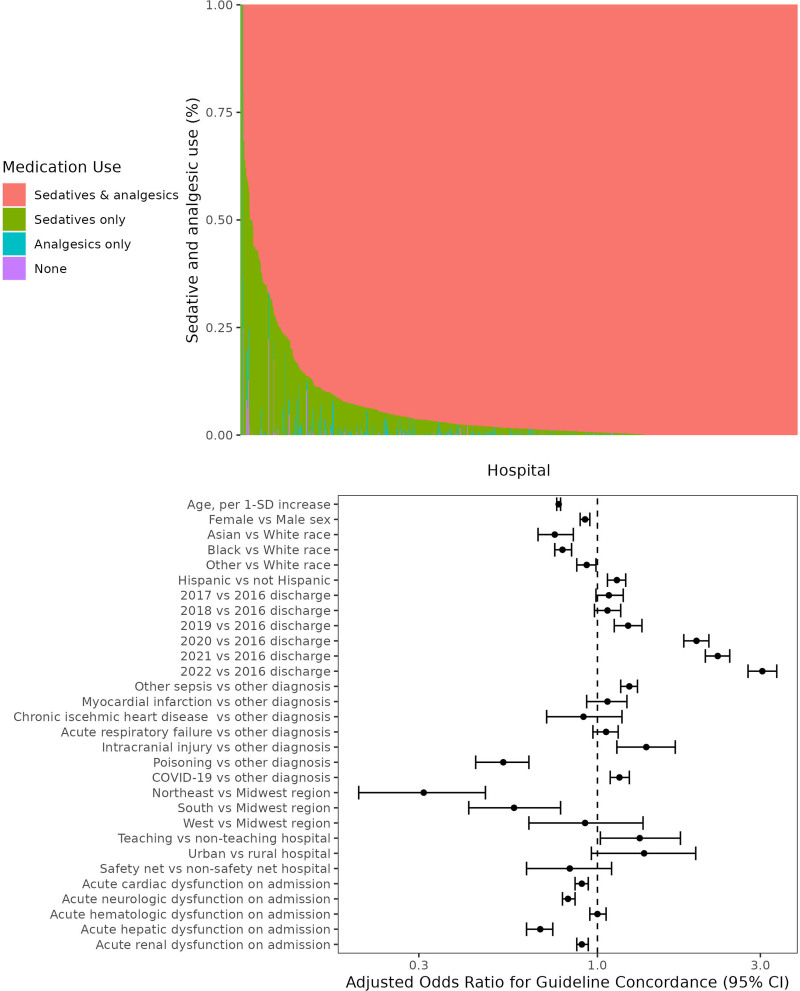
Sedation and analgesia practices during neuromuscular blockade. The **top** shows the percentage of patient-days (*y*-axis) at each hospital (*x*-axis) associated with use of each category of sedation and analgesia (both sedation and analgesia [i.e., guideline-concordant], just sedation, just analgesia, and neither sedation nor analgesia). Hospitals are ranked from lowest to highest use of guideline concordance. The **bottom** shows associations between fixed effects and guideline-concordant sedation and analgesia use during neuromuscular blockade from the hierarchical model. Associations are presented as adjusted odds ratios (*points*) and associated 95% CIs (*whiskers*). The adjusted odds ratio for the association between cerebral palsy principal diagnosis vs. other diagnosis (adjusted odds ratio of < 0.01 [95% CI, <0.001 to 0.01]) is not shown to improve clarity.

Among the 871 included hospitals, 856 (98.3%) used guideline-concordant sedation and analgesia on greater than or equal to 50% of patient-days, 10 (1.1%) used sedation-only on greater than or equal to 50% of patient-days, 1 (0.1%) used analgesia-only on greater than or equal to 50% of patient-days, and 4 (0.6%) had no predominant sedation and analgesia strategy (**Fig. 1**). After accounting for model mixed effects, the ICC (a measure of residual unexplained clustering by hospital) was 42.3%.

Strong predictors of guideline concordance included discharge year (2022 vs. 2016: aOR, 3.04 [95% CI, 2.76–3.35]; 2021 vs. 2016: aOR, 2.25 [95% CI, 2.07–2.44]; and 2020 vs. 2016: aOR, 1.95 [95% CI, 1.79–2.12]), principal diagnosis (intracranial injury vs. other diagnosis aOR, 1.39 [95% CI, 1.14–1.69] and sepsis vs. other diagnosis aOR, 1.24 [95% CI, 1.17–1.31]), and hospital teaching status (teaching vs. nonteaching aOR, 1.33 [95% CI, 1.02–1.75]; Fig. 1). Patient-days for older patients, female patients, patients with organ dysfunction, and patients identified as Black or Asian Race were less likely to have guideline-concordant sedation and analgesia.

## DISCUSSION

In a multicenter study of approximately 25% of U.S. hospital admissions, patients receiving sustained neuromuscular blockade generally received guideline-concordant parenteral sedation and analgesia. However, guideline-concordant practice was variable between hospitals and was associated with several clinical and demographic characteristics (suggesting clinicians may be withholding guideline-concordant sedation and analgesia in certain patients). Furthermore, no parenteral sedation was administered approximately 1% of the time. Thus, there remains a need to further investigate guideline concordance and reduce potentially inappropriate practice variation.

Limited data exist for sedation gaps in critically ill patients receiving prolonged neuromuscular blockade. Our finding of guideline-discordant practices in certain patient groups is aligned with known disparities in the care of ICU patients based on age, sex, and race (13, 14). We also found lower rates of compliance among patients with organ dysfunction, a finding we speculate is related to concerns regarding ineffective drug metabolism or assumptions regarding awareness in patients with hepatic or renal failure. In 16.5% of patient-days, dexmedetomidine may have been used alone as a sedative; however, dexmedetomidine achieves light or “arousable” sedation rather than deep sedation (15). Given the association of guideline-discordant practices with patient recall (16) and the known harm to patients who experience recall during paralysis (4), our findings highlight the need for multidisciplinary efforts focused on the identification of factors contributing to sedation and analgesia gaps.

Our study has several limitations, namely that use of charge codes rather than electronic medical record data does not allow for extraction of granular dosing and administration information from the database and limits our ability to precisely determine guideline concordance. Thus, our results should not be interpreted as evidence of awake paralysis. While we accounted for most commonly used parenteral sedatives and analgesics, use of alternative agents (e.g., inhaled anesthetics) was not captured. We did not have access to sedation or pain scales to determine the adequacy of sedation and analgesia; thus, our study may overestimate guideline concordance. We evaluated patient-days as either guideline-concordant or discordant and did not examine associations between patient- and hospital-factors and partial adherence to guidelines (e.g., sedation-only, analgesia-only). We were not able to distinguish between continuous and intermittent NMBA, sedation, or analgesia use, which may potentially under- or over-estimate guideline-concordant practices. Similarly, exclusion of the index day of NMBA prevents characterization of sedation gaps occurring at NMBA initiation. Finally, the low rate of discordance combined with the large sample size may increase risks of identifying spurious associations due to random error.

In a large multicenter cohort study, medication charge code data suggests guideline-concordant parenteral sedation and analgesia was used the majority of the time in critically ill patients receiving IMV and sustained neuromuscular blockade. However, we also identified potentially concerning variation in guideline concordance that warrant further investigation into ongoing trends in use of sedation and analgesia during NMB using more granular medication administration data.

## ACKNOWLEDGMENTS

We thank Dr. Christopher Kearney for his perspectives on study design.

## Supplementary Material
