# Supplementary material for: Guideline-Concordant Sedative and Analgesia Use in Critically Ill Patients Receiving Sustained Neuromuscular Blockade
Source: Crit Care Explor. 2026 Jan 26;8(2):e1370. doi: 10.1097/CCE.0000000000001370 (PMC12834453; doi:10.1097/CCE.0000000000001370)
Supplement: Supplementary file 1 [file cc9-8-e1370-s001.pdf]

SUPPLEMENTAL DIGITAL CONTENT

Guideline-concordant sedative and analgesia use in critically ill patients receiving sustained neuromuscular blockade

Megan E. Feeney, PharmD, BCCCP[1], Ava E. Cascone, PharmD, BCCCP[2], Anica C. Law, MD, MS[3], Allan J. Walkey, MD, MSc[4,5], Nicholas A. Bosch, MD, MSc[3,6]

[1]Department of Pharmacy, Boston Medical Center, Boston, MA

[2]Department of Pharmacy, Penn Presbyterian Medical Center, Philadelphia, PA

[3]The Pulmonary Center, Boston University Chobanian & Avedisian School of Medicine, Boston, MA

[4]Department of Healthcare Delivery and Population Sciences, University of Massachusetts Chan Medical School - Baystate, Springfield, Massachusetts.

[5]Division of Health Systems Science, Department of Medicine, University of Massachusetts Medical School, Worcester, Massachusetts.

[6]Evans Center for Implementation and Improvement Science, Department of Medicine, Boston University Chobanian & Avedisian School of Medicine, Boston, MA

Table of Contents

Pages 3-7	eTable 1: Included Charge Codes and Expected Durations of Each Formulation as Extrapolated from the ACURASYS Trial
Page 8	eTable 2: Infusion Rate Derivations for Included Medication Charge Codes
Pages 9-10	eTable 3: Patient Characteristics Stratified by Sedation and Analgesia Strategy during Neuromuscular Blockade

eTable 1. Included Charge Codes and Expected Durations of Each Formulation as Extrapolated from the ACURASYS Trial

Charge Code Description	mg per dose unit[a]	T_ACURASYS[b] (hours)	Total number of patient-days before extrapolation	Percent patient-days before extrapolation
*DIAZEPAM, VALIUM INJ 10MG 2ML	10	0.3	19	0.01%
DIAZEPAM, VALIUM INJ 5MG 1ML	5	0.1	583	0.16%
DIAZEPAM, VALIUM VL 5MG/ML 10ML	50	1.5	3	0.00%
*DIAZEPAM, VALIUM VL 5MG/ML 1ML	5	0.1	4	0.00%
DIAZEPAM, VALIUM VL 5MG/ML 2ML	10	0.3	274	0.08%
DIAZEPAM, VALIUM VL 5MG/ML 5ML	25	0.7	2963	0.82%
ETOMIDATE, AMIDATE VL 2MG/ML 10ML	20	0.7	2963	0.82%
ETOMIDATE, AMIDATE VL 2MG/ML 1ML	2	0.1	212	0.06%
ETOMIDATE, AMIDATE VL 2MG/ML 20ML	40	1.3	2615	0.72%
FENTANYL, SUBLIMAZE AMP 0.05MG/ML 10ML	0.5	3.3	1279	0.35%
FENTANYL, SUBLIMAZE AMP 0.05MG/ML 1ML	0.05	0.3	8169	2.25%
FENTANYL, SUBLIMAZE AMP 0.05MG/ML 20ML	1	6.7	17652	4.86%
FENTANYL, SUBLIMAZE AMP 0.05MG/ML 2ML	0.1	0.7	178816	49.21%
FENTANYL, SUBLIMAZE AMP 0.05MG/ML 40ML	2	13.3	3889	1.07%
FENTANYL, SUBLIMAZE AMP 0.05MG/ML 5ML	0.25	1.7	3122	0.86%
FENTANYL, SUBLIMAZE VL 0.05MG/ML 30ML	1.5	10.0	456	0.13%
FENTANYL, SUBLIMAZE VL 0.05MG/ML 50ML	2.5	16.7	42209	11.62%
*HYDROMORPHONE, DILAUDID AMP 1MG/ML 1ML	1	0.5	5	0.00%
*HYDROMORPHONE, DILAUDID AMP 4MG/ML 1ML	4	1.8	0	0.00%
HYDROMORPHONE, DILAUDID HP AMP 10MG/ML 1ML	10	4.5	1158	0.32%
HYDROMORPHONE, DILAUDID HP AMP 10MG/ML 5ML	50	22.7	5258	1.45%
HYDROMORPHONE, DILAUDID INJ 1MG/ML 1ML	1	0.5	7093	1.95%
HYDROMORPHONE, DILAUDID INJ 2MG/ML 1ML	2	0.9	3140	0.86%
HYDROMORPHONE, DILAUDID INJ 3MG/ML 1ML	3	1.4	2	0.00%
HYDROMORPHONE, DILAUDID INJ 4MG/ML 1ML	4	1.8	15849	4.36%
HYDROMORPHONE, DILAUDID VL 10MG/ML 2ML	20	9.1	268	0.07%

Charge Code Description	mg per dose unit[a]	T_ACURASYS[b] (hours)	Total number of patient-days before extrapolation	Percent patient-days before extrapolation
HYDROMORPHONE, DILAUDID VL 10MG/ML 50ML	500	227.3	526	0.14%
*HYDROMORPHONE, DILAUDID VL 2MG/ML 1ML	2	0.9	22	0.01%
HYDROMORPHONE, DILAUDID VL 2MG/ML 20ML	40	18.2	175	0.05%
KETAMINE, KETALAR VL 100MG/ML 1ML	100	0.4	1804	0.50%
KETAMINE, KETALAR VL 100MG/ML 5ML	500	2.0	9139	2.51%
KETAMINE, KETALAR VL 10MG/ML 1ML	10	0.0	994	0.27%
KETAMINE, KETALAR VL 10MG/ML 20ML	200	0.8	1640	0.45%
KETAMINE, KETALAR VL 10MG/ML 25ML	250	1.0	109	0.03%
KETAMINE, KETALAR VL 10MG/ML 50ML	500	2.0	9	0.00%
KETAMINE, KETALAR VL 50MG/ML 10ML	500	2.0	15347	4.22%
KETAMINE, KETALAR VL 50MG/ML 1ML	50	0.2	2817	0.78%
LORAZEPAM, ATIVAN INJ 1MG	1	0.2	255	0.07%
*LORAZEPAM, ATIVAN INJ 2MG	2	0.4	364	0.10%
*LORAZEPAM, ATIVAN INJ 4MG	4	0.9	11	0.00%
LORAZEPAM, ATIVAN VL 2MG/ML 10ML	20	4.4	845	0.23%
LORAZEPAM, ATIVAN VL 2MG/ML 1ML	2	0.4	17574	4.84%
LORAZEPAM, ATIVAN VL 4MG/ML 10ML	40	8.9	690	0.19%
LORAZEPAM, ATIVAN VL 4MG/ML 1ML	4	0.9	57	0.02%
MIDAZOLAM, VERSED INJ 10MG	10	1.1	1417	0.39%
MIDAZOLAM, VERSED VL 1MG/ML 10ML	10	1.1	6594	1.81%
MIDAZOLAM, VERSED VL 1MG/ML 1ML	1	0.1	149202	41.06%
MIDAZOLAM, VERSED VL 1MG/ML 2ML	2	0.2	10067	2.77%
MIDAZOLAM, VERSED VL 1MG/ML 5ML	5	0.6	1354	0.37%
MIDAZOLAM, VERSED VL 5MG/ML 10ML	50	5.6	23277	6.41%
MIDAZOLAM, VERSED VL 5MG/ML 1ML	5	0.6	2429	0.67%
MIDAZOLAM, VERSED VL 5MG/ML 2ML	10	1.1	693	0.19%
MIDAZOLAM, VERSED VL 5MG/ML 5ML	25	2.8	585	0.16%
MORPHINE AMP 1MG/ML 2ML	2	0.1	54	0.01%

Charge Code Description	mg per dose unit[a]	T_ACURASYS[b] (hours)	Total number of patient-days before extrapolation	Percent patient-days before extrapolation
MORPHINE AMP 1MG/ML 30ML	30	2.0	17	0.00%
MORPHINE AMP 1MG/ML 60ML	60	4.1	5	0.00%
*MORPHINE AMP 2MG/ML 1ML	2	0.1	0	0.00%
MORPHINE INJ 10MG/ML 1ML	10	0.7	2316	0.64%
MORPHINE INJ 10MG/ML 2ML	20	1.4	0	0.00%
MORPHINE INJ 15MG/ML 1ML	15	1.0	6	0.00%
MORPHINE INJ 15MG/ML 2ML	30	2.0	0	0.00%
MORPHINE INJ 25MG/ML 10ML	250	16.9	65	0.02%
MORPHINE INJ 25MG/ML 20ML	500	33.8	5	0.00%
MORPHINE INJ 25MG/ML 40ML	1000	67.6	0	0.00%
MORPHINE INJ 25MG/ML 4ML	100	6.8	218	0.06%
MORPHINE INJ 2MG/ML 1ML	2	0.1	2030	0.56%
MORPHINE INJ 2MG/ML 2ML	4	0.3	0	0.00%
MORPHINE INJ 4MG/ML 1ML	4	0.3	1553	0.43%
MORPHINE INJ 4MG/ML 2ML	8	0.5	0	0.00%
MORPHINE INJ 50MG/ML 20ML	1000	67.6	332	0.09%
MORPHINE INJ 8MG/ML 1ML	8	0.5	32	0.01%
MORPHINE INJ 8MG/ML 2ML	16	1.1	0	0.00%
MORPHINE VL 10MG/ML 10ML	100	6.8	46	0.01%
*MORPHINE VL 10MG/ML 1ML	10	0.7	32	0.01%
*MORPHINE VL 15MG/ML 1ML	15	1.0	0	0.00%
MORPHINE VL 15MG/ML 20ML	300	20.3	11	0.00%
MORPHINE VL 1MG/ML 100ML	100	6.8	135	0.04%
MORPHINE VL 2MG/ML 60ML	120	8.1	0	0.00%
MORPHINE VL 3MG/ML 50ML	150	10.1	0	0.00%
*MORPHINE VL 50MG/ML 20ML	1000	67.6	0	0.00%
MORPHINE VL 5MG/ML 1ML	5	0.3	33	0.01%
MORPHINE VL 5MG/ML 30ML	150	10.1	4	0.00%

Charge Code Description	mg per dose unit[a]	T_ACURASYS[b] (hours)	Total number of patient-days before extrapolation	Percent patient-days before extrapolation
*MORPHINE VL 8MG/ML 1ML	8	0.5	0	0.00%
MORPHINE, DURAMORPH AMP 0.5MG/ML 10ML	5	0.3	1	0.00%
MORPHINE, DURAMORPH AMP 0.5MG/ML 2ML	1	0.1	0	0.00%
MORPHINE, DURAMORPH AMP 1MG/ML 10ML	10	0.7	94	0.03%
MORPHINE AMP 10MG/ML 20ML	200	13.5	103	0.03%
*MORPHINE AMP 25MG/ML 20ML	500	33.8	0	0.00%
PENTOBARBITAL, NEMBUTAL INJ 50MG/ML 1ML	50	0.2	505	0.14%
PENTOBARBITAL, NEMBUTAL INJ 50MG/ML 2ML	100	0.4	21	0.01%
PENTOBARBITAL, NEMBUTAL VL 50MG/ML 20ML	1000	4.4	104	0.03%
*PENTOBARBITAL, NEMBUTAL VL 50MG/ML 2ML	100	0.4	0	0.00%
PENTOBARBITAL, NEMBUTAL VL 50MG/ML 50ML	2500	11.0	56	0.02%
*PHENOBARB AMP 130MG/ML 1ML	130	1.9	0	0.00%
PHENOBARB INJ 130MG/ML 1ML	130	1.9	1125	0.31%
PHENOBARB INJ 30MG/ML 1ML	30	0.4	2	0.00%
PHENOBARB INJ 60MG/ML 1ML	60	0.9	1218	0.34%
PHENOBARB VL 65MG/ML 1ML	65	1.0	1479	0.41%
PROPOFOL, DIPRIVAN AMP 10MG/ML 10ML	100	1.0	1355	0.37%
PROPOFOL, DIPRIVAN AMP 10MG/ML 20ML	200	2.0	1356	0.37%
PROPOFOL, DIPRIVAN VL 10MG/ML 100ML	1000	10.0	75892	20.88%
PROPOFOL, DIPRIVAN VL 10MG/ML 1ML	10	0.1	135604	37.32%
PROPOFOL, DIPRIVAN VL 10MG/ML 50ML	500	5.0	1173	0.32%
FOSPROPOFOL, LUSEDRA VL 35MG/ML 30ML	1050	5.6	0	0.00%
KETAMINE INJ 0.9% NACL 50MG/5ML	250	1.0	112	0.03%
HYDROMORPHONE, DILAUDID INJ 0.5MG/0.5ML	0.5	0.2	1179	0.32%
KETAMINE, KETALAR VL 100MG/ML 10ML	1000	4.1	1215	0.33%
MIDAZOLAM 1MG/ML 100ML NS	100	11.1	22508	6.19%
MIDAZOLAM 1MG/ML 50ML NS	50	5.6	6007	1.65%

**Charge Code Description**	**mg per dose unit[a]**	**T_ACURASYS[b] (hours)**	**Total number of patient-days before extrapolation**	**Percent patient-days before extrapolation**
MIDAZOLAM (WG CRITICAL CARE-NTE) VL 1MG/ML 1ML	1	0.1	0	0.00%
MORPHINE (FRESENIUS KABI-NTE) INJ 10MG/ML 1ML	10	0.7	0	0.00%

[a] The maximum calculated mg dose that could have been administered from 1 unit of that charge code (example: lorazepam 4 mg/mL 10 mL vial = 40 mg)

[b] The potential duration (in hours) which that mg dose could have lasted if run at the rate determined from the average sedative and analgesia rates received by patients in the first 48 hours in the ACURASYS(1) trial.

[c] To calculate the total potential duration of a given charge code on a given calendar day, the potential duration was multiplied by the number of charges for that medication and then rounded up to the nearest 24 hours to account for calendar day granularity. For example, a patient with 3 charges for lorazepam 4mg/mL 10 mL vial on a given calendar day would have a potential duration of 8.9 hours x 3 charges = 26.7 hours, rounding up to 48 hours. Thus, the patient would be assigned as having received sedation on the day of the lorazepam charges and for the next two calendar days.

eTable 2. Infusion Rate Derivations for Included Medication Charge Codes

Medication	Rate from or equivalent to ACURASYS (mg/hr)	
Fentanyl	0.15	Converted(2, 3) from Sufentanil doses in ACURASYS(1)
Hydromorphone	2.2	Converted(2, 3) from Sufentanil doses in ACURASYS(1)
Morphine	14.8	Converted(2, 3) from Sufentanil doses in ACURASYS(1)
Ketamine	245.5	Supplement Table 9 - ACURASYS(1)
Midazolam	9	Supplement Table 9 - ACURASYS(1)
Lorazepam	4.5	Converted(4) from midazolam doses in ACURASYS(1)
Diazepam	34	Converted(5) from midazolam doses in ACURASYS(1)
Propofol	100	Supplement Table 9 - ACURASYS(1)
Dexmedetomidine	0.06	Used dose of 1.2 mcg/kg/hr
Phenobarbital	68	Converted(5) from midazolam doses in ACURASYS(1)
Pentobarbital	226.7	Used conversion factor of 0.3 (6)
Etomidate	30	Used high dose 0.6 mg/kg
Fospropofol	186	Converted 1.86 mg fospropofol: 1 mg propofol (7)
*All weight-based doses were converted using a weight of 50 kg<br>** All opioid conversions used a 25% cross tolerance correction		

1. Papazian L, Forel J-M, Gacouin A, et al.: Neuromuscular blockers in early acute respiratory distress syndrome. *N Engl J Med* 2010; 363:1107–1116

2. Patanwala AE, Duby J, Waters D, et al.: Opioid conversions in acute care. *Ann Pharmacother* 2007; 41:255–266

3. Opioid (Opiate) Equianalgesia Conversion Calculator - ClinCalc.com [Internet]. [cited 2025 Oct 7] Available from: https://clincalc.com/opioids/

4. Barr J, Zomorodi K, Bertaccini EJ, et al.: A double-blind, randomized comparison of i.v. lorazepam versus midazolam for sedation of ICU patients via a pharmacologic model. *Anesthesiology* 2001; 95:286–298

5. Benzodiazepine Equivalents Conversion Calculator - ClinCalc.com [Internet]. [cited 2025 Oct 7] Available from: https://clincalc.com/benzodiazepine/

6. Figure 4-6, Other Sedative-Hypnotics and Their Phenobarbital Withdrawal Equivalents [Internet]. 2006; [cited 2025 Oct 7] Available from: https://www.ncbi.nlm.nih.gov/books/NBK64116/

7. Bengalorkar GM, Bhuvana K, Sarala N, et al.: Fospropofol: Clinical Pharmacology. *J Anaesthesiol Clin Pharmacol* 2011; 27:79–83

eTable 3. Patient Characteristics Stratified by Sedation and Analgesia Strategy during Neuromuscular Blockade

Characteristic	Sedation and Analgesia Strategy				
	Overall (n=363,382)	Both sedation and analgesia (n=345,660)	Sedation Only (n=15,618)	Analgesia Only (n=1,348)	Neither sedation nor analgesia (n=756)
Age in years, mean (standard deviation)	55 (14)	55 (14)	58 (14)	58 (14)	60 (15)
Sex, No. (%)					
Female	137077 (37.7)	130125 (37.6)	6074 (38.9)	578 (42.9)	300 (39.7)
Male	226194 (62.2)	215429 (62.3)	9539 (61.1)	770 (57.1)	456 (60.3)
Race, No. (%)					
Asian	8963 (2.5)	8387 (2.4)	497 (3.2)	35 (2.6)	44 (5.8)
Black	54060 (14.9)	51165 (14.8)	2530 (16.2)	275 (20.4)	90 (11.9)
Other	42334 (11.6)	39658 (11.5)	2508 (16.1)	117 (8.7)	51 (6.7)
Unknown	18669 (5.1)	17124 (5.0)	1270 (8.1)	137 (10.2)	138 (18.3)
White	239356 (65.9)	229326 (66.3)	8813 (56.4)	784 (58.2)	433 (57.3)
Hispanic ethnicity, No. (%)					
Yes	74656 (20.5)	71396 (20.7)	2800 (17.9)	318 (23.6)	142 (18.8)
No	244691 (67.3)	232623 (67.3)	10757 (68.9)	803 (59.6)	508 (67.2)
Unknown	44035 (12.1)	41641 (12.0)	2061 (13.2)	227 (16.8)	106 (14.0)
Discharge year, No. (%)					
2016	18116 (5.0)	16890 (4.9)	1080 (6.9)	84 (6.2)	62 (8.2)
2017	19169 (5.3)	18023 (5.2)	1059 (6.8)	55 (4.1)	32 (4.2)
2018	20169 (5.6)	18832 (5.4)	1206 (7.7)	107 (7.9)	24 (3.2)
2019	21036 (5.8)	19718 (5.7)	1201 (7.7)	92 (6.8)	25 (3.3)
2020	82836 (22.8)	78138 (22.6)	3939 (25.2)	483 (35.8)	276 (36.5)
2021	159056 (43.8)	152500 (44.1)	5823 (37.3)	452 (33.5)	281 (37.2)
2022	43000 (11.8)	41559 (12.0)	1310 (8.4)	75 (5.6)	56 (7.4)
Principal diagnosis[a], No. (%)					
Other sepsis (A41.x)	141564 (39.0)	134689 (39.0)	6054 (38.8)	558 (41.4)	263 (34.8)
COVID-19 (U07.1)	132261 (36.4)	126406 (36.6)	5054 (32.4)	438 (32.5)	363 (48.0)
Respiratory failure (J96.x)	14714 (4.0)	13862 (4.0)	796 (5.1)	36 (2.7)	20 (2.6)
Intracranial injury (S06.x)	5396 (1.5)	5274 (1.5)	112 (0.7)	2 (0.1)	8 (1.1)
Acute myocardial infarction (I21.x)	4739 (1.3)	4357 (1.3)	331 (2.1)	46 (3.4)	5 (0.7)
Poisoning[b] (T40.x)	2069 (0.6)	1886 (0.5)	171 (1.1)	9 (0.7)	3 (0.4)
Chronic ischemic heart disease (I25.x)	1268 (0.3)	1184 (0.3)	63 (0.4)	20 (1.5)	1 (0.1)

Cerebral palsy (G80.x)	13 (0.0)	2 (0.0)	2 (0.0)	0 (0.0)	9 (1.2)
Other	61358 (16.9)	58000 (16.8)	3035 (19.4)	239 (17.7)	84 (11.1)
Acute organ dysfunction on admission, No. (%)					
Cardiac	106787 (29.4)	100929 (29.2)	5162 (33.1)	470 (34.9)	226 (29.9)
Neurologic	57289 (18.8)	53935 (15.6)	3043 (19.5)	211 (15.7)	100 (13.2)
Hematologic	45803 (12.6)	43506 (12.6)	1962 (12.6)	242 (18.0)	93 (12.3)
Hepatic	12171 (3.3)	11327 (3.3)	732 (4.7)	79 (5.9)	33 (4.4)
Renal	133078 (36.6)	125657 (36.4)	6530 (41.8)	571 (42.4)	320 (42.3)
Teaching hospital, No. (%)	195886 (53.9)	187260 (54.2)	7390 (47.3)	740 (54.9)	496 (65.6)
Urban hospital, No. (%)	327632 (90.2)	312112 (90.3)	13789 (88.3)	1191 (88.4)	540 (71.4)
Safety net hospital, No. (%)	108456 (29.8)	101910 (29.5)	5694 (36.5)	614 (45.5)	238 (31.5)

[a]The five most common principal diagnoses (based on International Classification of Diseases, Tenth Revision Discharge Diagnosis Codes) within each sedation and analgesia strategy were included. Since not all strategies had the same five most common diagnoses, eight total principal diagnoses were included. All other principal diagnoses were included under the category of "other".

[b]Poisoning by, adverse effect of and underdosing of narcotics and psychodysleptics

[c]Not included in models
